# Improving Pain Management at the Nursing Education Level: Evaluating Knowledge and Attitudes

**Published:** 2014-01-01

**Authors:** Jessica Latchman

## Abstract

Unmanaged pain is a prevalent problem faced by many cancer patients. One part of this problem centers on a lack of emphasis on pain management in the undergraduate nursing curriculum. This study examined the knowledge and attitudes of 41 undergraduate nursing students regarding pain management. Students voluntarily completed a demographic data form, the Nurses’ Attitude Survey, and the Pain Management Principles Assessment Tool. A mean score of 19.4 out of a possible 31 was achieved on the knowledge test, whereas a mean score of 17.0 was achieved on the Nurses’ Attitude Survey. A weak-to-moderate relationship between knowledge and attitudes was found. Although students had positive attitudes regarding pain management, many still lacked the fundamental knowledge essential for adequately managing pain. The sample size was relatively small and not demographically diverse, but the response from the sample was sufficient to provide statistically meaningful data. In the quest to improve patient outcomes, these findings suggest the need to develop specific strategies to effectively teach undergraduate nursing students about pain management.

Unmanaged pain has been identified as a major barrier in the overall care of the oncology patient. In fact, more than 70% of this population will experience chronic cancer-related pain at some point in the course of their disease, with the majority receiving ineffective treatment. Cancer pain—which can be caused by tissue damage due to tumor burden or by treatments such as radiation therapy and chemotherapy—can have devastating effects on the quality of life of patients and their caregivers (American Cancer Society, 2009). Consequently, guidelines for pain management in oncology have been developed to foster better assessment techniques and interventions (American Society of Clinical Oncology, 2007).

Pain has the potential to affect all levels of psychophysiologic capabilities, including maintaining relationships with others, carrying out the activities of daily living, and performing at work. From a financial perspective, chronic pain costs an estimated $90 billion in economic resources. This can be a result of lost time from work, disability, and reduced productivity (Porter & Keefe, 2011). Pain is also associated with many psychological symptoms such as depression, mood, and anxiety disorders, and it affects patients’ overall quality of life (Turks, 2006; Porter & Keefe, 2011).

Previous studies have demonstrated a lack of emphasis on pain management in the undergraduate nursing curriculum, but few studies have been conducted during the past 20 years. The aim of this study was to explore the current knowledge and attitudes on pain management among nursing students as they finish their educational program and prepare to enter the clinical arena.

## Review of the Literature

A lack of knowledge as a major barrier to effective pain management was reported in studies from the early 1990s and is clearly still evident in more recent literature (Diekmann & Wassem, 1991; Chiu, Trinca, Lim, and Tuazon, 2003; Goodrich, 2006). Numerous gaps were also seen in nursing programs that had little or no content on cancer pain management, hindering students’ ability to learn effective pain management (Diekmann & Wassem, 1991; Goodrich, 2006). Deficiencies were noted in areas such as the physiology of pain, assessment parameters, differentiation of addiction from tolerance and physical dependence, and understanding the importance of the patient’s self-report as the best indicator of his or her own pain. The message sent by these researchers to schools of nursing was clear: Provide more content on pain management (Rieman & Gordon, 2007; Bernardi, Catania, Lambert, Tridello, & Luzzani, 2007).

Individual attitudes and personal biases can also influence pain management in a variety of ways. A patient exhibiting a cheerful attitude with no outward signs of physical or emotional distress may not be prescribed or given adequate doses of pain medication, despite being in severe pain (McMillan, Tittle, Hagan, Laughlin, & Tabler, 2000). The advanced practitioner (AP) or nurse may assume that with little or no visible sign of pain, the patient may not be experiencing much pain and may not require pain medication. Concerns of addiction may also deter APs and nurses from administering opioid analgesics (McMillan, Tittle, Hagan, & Small, 2005; Rushton, Eggett, & Sutherland, 2003; Ferrell, McGuire, & Donovan, 1993; Lasch et al., 2002; McMillan et al., 2000).

Continuing education, updated with current treatment guidelines, and the implementation of new educational strategies may help to adequately prepare future practitioners and nurses to manage pain more effectively (Lasch et al., 2002; McMillan et al., 2005; Wilkes, Lasch, Lee, Greenhill, & Chiri, 2003).

## Methods

**Sample** 

For this descriptive, cross-sectional study, we surveyed a convenience sample of undergraduate students pursuing a Bachelor of Science degree in Nursing (BSN) at a large research university in the southeastern United States. To be eligible for the study, students had to be in their final year of the program and had to have completed the pharmacology and pathophysiology courses in which the majority of pain management content is delivered. The sample size was estimated using power analytic techniques. With a power of 0.80 and an á set 0.05 for a Pearson correlation, a sample size of 30 was determined to be an adequate number to detect statistical significance.

**Instruments** 

The instruments used in this study were the Nurses’ Attitude Survey (NAS) and the Pain Management Principles Assessment Test (PMPAT) as well as a demographic data questionnaire.

**Nurses’ Attitude Survey** 

The NAS, created by McMillan and colleagues (2000), is a 25-item instrument that uses a four-point Likert-type format to assess attitudes toward pain management. Responses for the instrument ranged from strongly disagree to strongly agree, with raw scores varying from 1 to 4 for each item. The higher the score, the more positive the attitudes held by respondents. Internal consistency reliability was found using Cronbach’s á (r = 0.70). Validity was demonstrated after it was pre- and posttested among nursing students (with a significant difference of t = 6.88, *p* < .01; McMillan et al., 2000).

**Pain Management Principles Assessment Test** 

The PMPAT is a 31-item multiple-choice test with 4 response choices per question. The questionnaire was designed to test pain management knowledge. Scores for the survey ranged from 0 to 31, or 0% to 100%, with higher scores meaning more questions were answered correctly. The tool was designed based on a blueprint from previous research studies attesting to its content validity. Validity was found to be significantly high from pre- to post-test (t = 6.76, *p* < .01). Reliability was also discovered to be significantly high (r = 0.84,* p* = .00; McMillan et al., 2000).

**Demographic Data Questionnaire** 

Each participant was asked to complete a demographic data questionnaire. The form incorporated questions on age, gender, ethnicity, current semester in the BSN program, highest level of education achieved, work experience, current work status, and previous training in pain management (Table 1).

**Table 1:**
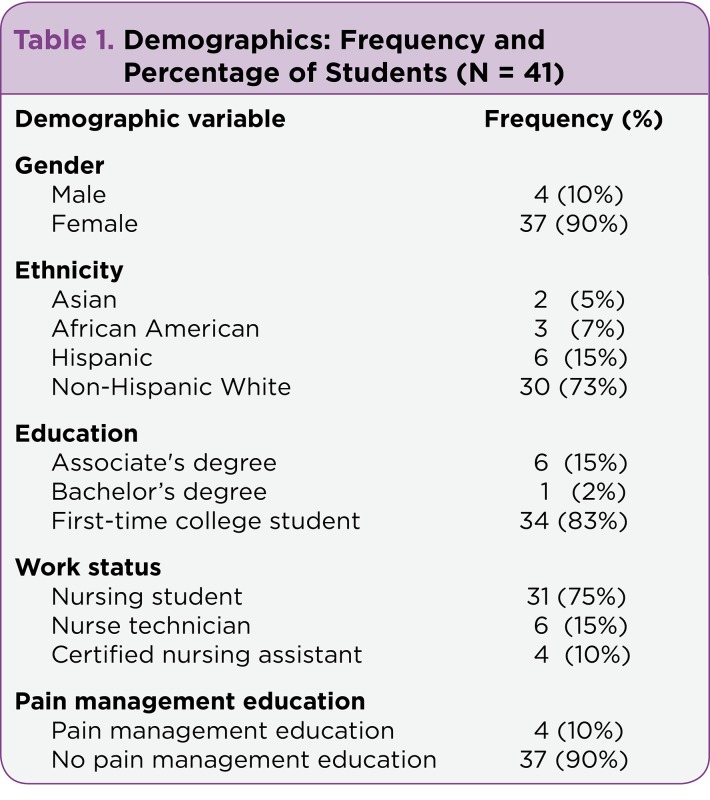
Table 1. Demographics: Frequency and Percentage of Students (N = 41)

## Procedure

The study was approved by the Institutional Review Board. The questionnaire/survey was given to each student during a class period without the presence of the instructor and with no coercion by the research team. A brief explanation was given regarding the study, and students were given the opportunity to ask pertinent questions.

## Data Analysis

Data were analyzed using descriptive statistics, including frequency, percentage, mean, standard deviation (SD), and Pearson correlation. The data were calculated using Microsoft Excel and the Statistical Package for Social Sciences (SPSS).

## Results

The sample consisted of 41 undergraduate nursing students in their final year of study. The majority were white, non-Hispanic women, 18 to 42 years old. A total of 10% (n = 4) of the students had participated in additional pain management training, whereas 90% (n = 37) had no training beyond that provided in their nursing program (Table 1). The mean score on the PMPAT knowledge subscale was 19.4 (SD = 3.0) out of 31 items, or 63%. If a passing score of 70% was used, 17% (n = 7) of students passed the pain management knowledge test (Table 2). Students scored 39% or less in areas of pain physiology: (1) pharmacology of pain medications; (2) appropriate time to medicate for pain; and (3) use of cutaneous stimulations as a measure of pain relief and total pain relief as the main goal of pain management practices. Subject areas in which students scored highest (93% to 100%) follow: (1) patients as the most accurate and reliable judge of their own pain; (2) accurate definition of tolerance; (3) patients should be in charge of their own pain management regimen; and (4) distraction as an approach to pain management (Table 3).

**Table 2:**
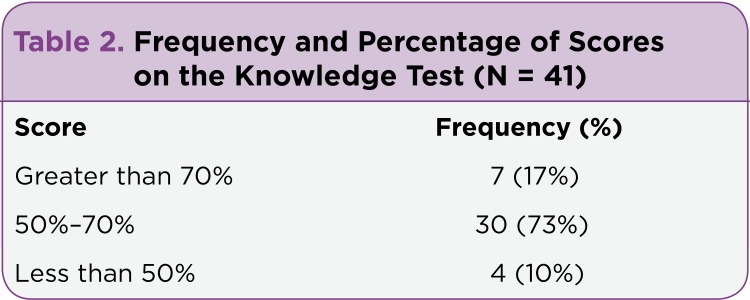
Table 2. Frequency and Percentage of Scores on the Knowledge Test (N = 41)

**Table 3:**
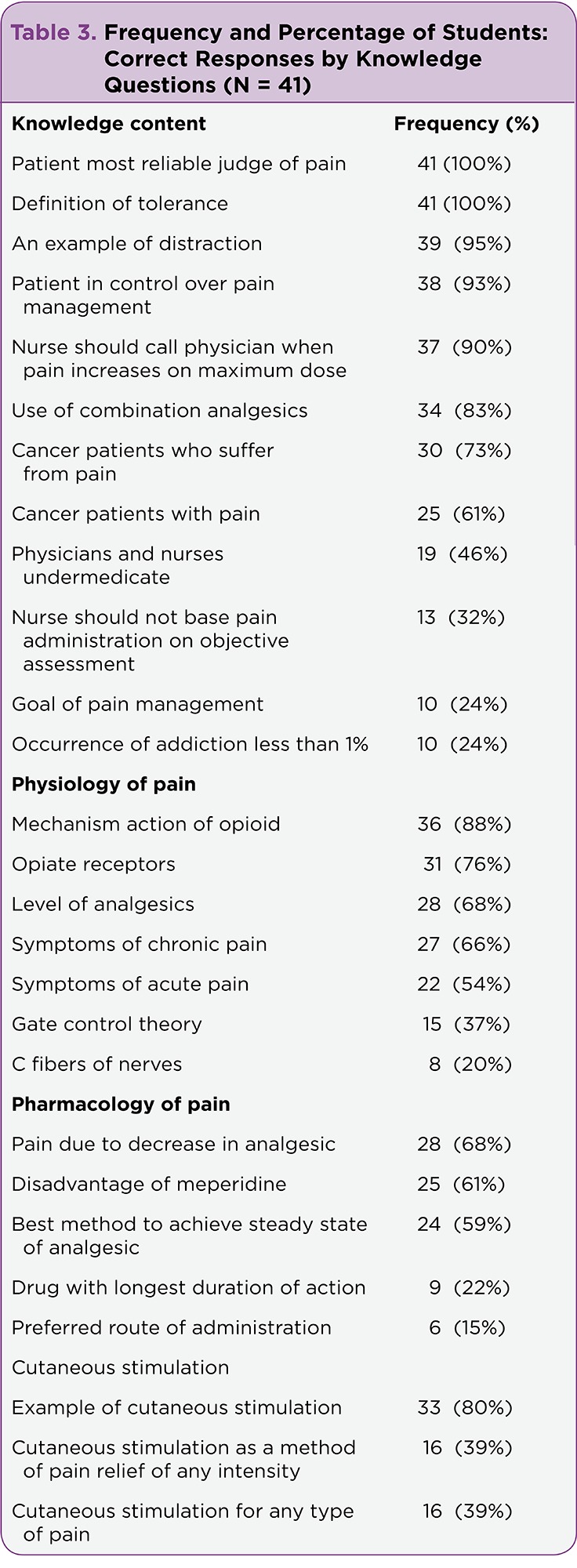
Table 3. Frequency and Percentage of Students: Correct Responses by Knowledge Questions (N = 41)

Scores from the attitude subscale included a mean score of 17.1 (SD = 2.6), with a range of 48% to 88% of students reporting positive attitudes toward pain management, depending on the item analyzed. Item analysis of the NAS indicated that the majority of students agreed that (1) distraction and diversion could decrease patients’ pain level, (2) lack of pain expression does not mean lack of pain, and (3) continuous assessment of pain and medication effectiveness is necessary for good pain management. Students had low scores in pain assessment, dosing of as-needed medications, and use of around-the-clock dosing (Table 4). A weak-to-moderate correlation between knowledge and attitudes was present (r = 0.33,* p* = .038).

**Table 4:**
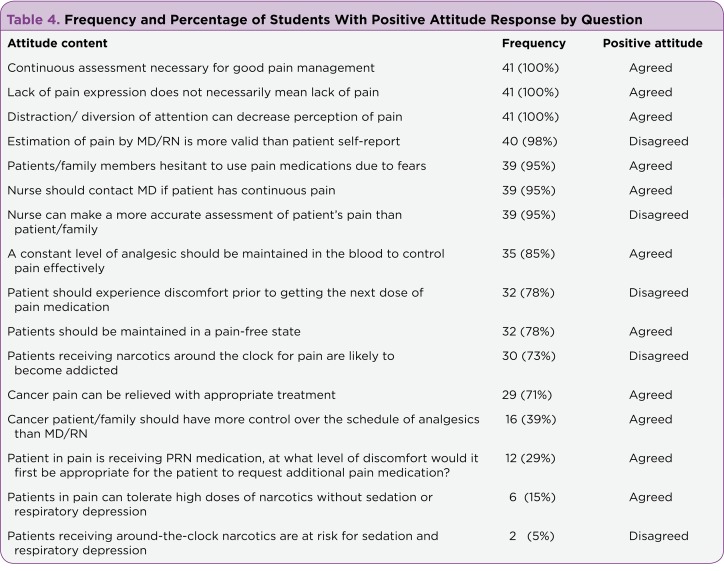
Table 4. Frequency and Percentage of Students With Positive Attitude Response by Question

## Discussion

The overall scores obtained from this study indicate that BSN-prepared students nearing graduation had minimal knowledge of basic pain management principles. This finding coincides with previous research studies done over the past 2 decades (Plaisance & Logan, 2006; Rushton et al., 2003; Ferrell et al., 1993; Lasch et al., 2002; McMillan et al., 2000). The lack of understanding of the basic pain management principles may hinder nurses’ ability to adequately manage pain upon graduation.

Participants had consistently low scores on the overall goal of pain management, the most appropriate time to administer pain medication, the preferred route of medication administration, the duration of action of methadone, the analgesic ceiling dose, and the use of nonpharmacologic techniques such as cutaneous stimulation in pain management. These findings demonstrate that pain-related content in the current curriculum had not been sufficient in meeting the needs of these BSN students. Therefore, if BSN-prepared nurses are inadequately prepared, it can be assumed that advance-practice nurses may also lack adequate pain management education at the graduate level. Thus, practitioners with inadequate pain management education are unable to provide appropriate care to patients in pain, especially in oncology, where pain management is a vital component of care.

Students were noted to have poor attitudes concerning patients receiving around-the-clock opioids and their ability to tolerate high doses of opioids without adverse effects. Students were not aware that sedation and respiratory depression rarely occur in patients with high opiate tolerance. These results were similar to those in previous studies and clearly indicate little improvement in this area over the past decade (McMillan et al., 2000). Therefore, if nurses developed a better understanding of the physiology of pain and the pharmacology of analgesics, a more positive attitude regarding pain management would emerge. This process could facilitate better relationships with patients and result in better patient outcomes in the long run.

## Relationship Between Knowledge and Attitudes

Students who generally had high scores on the knowledge test had corresponding high scores on the attitude test as well. However, there seems to be some discrepancy between similar questions on the knowledge survey and attitude questionnaire. Many students accurately stated that additional pain medication on an as-needed schedule should be administered before pain returns on the knowledge questionnaire. However, on the attitude survey, students strongly agreed that patients should experience discomfort prior to receiving the next dose of pain medication. These discrepancies may indicate that although attitudes affect the way in which pain is treated, most students still lack the fundamental knowledge and rationale for good pain management practices.

Students also had difficulty with the subject of addiction. Even though the majority could accurately define tolerance, only a small percentage of students were aware that patients with cancer are unlikely to become addicted to pain medication (see Table 5). Students also did not know that patients who received around-the-clock opioids for cancer pain are even less likely to become addicted. Therefore, we see that on the subject of addiction, both knowledge and attitude scores seem to be unrelated.

**Table 5:**
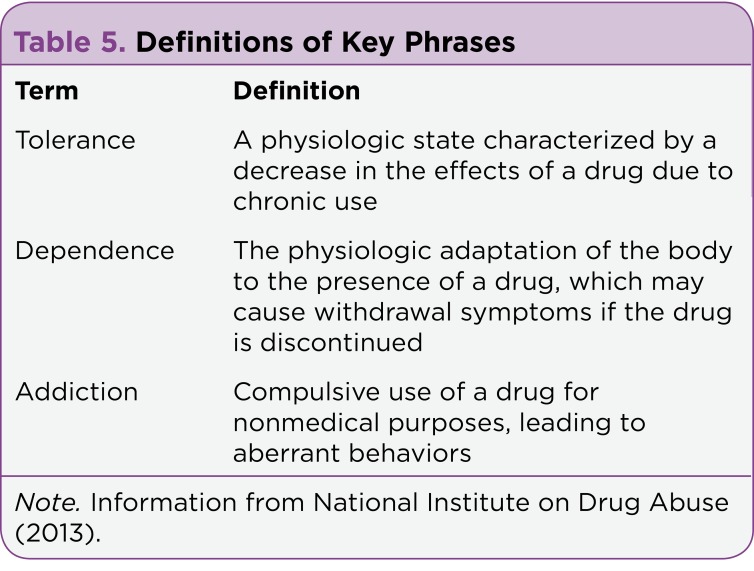
Table 5. Definitions of Key Phrases

Addiction and the recent increase in the trend of illegal diversion of prescription pain medication have become controversial issues. This scenario has affected the attitudes of APs and physicians with respect to prescribing opioids (Lin, Alfandre, & Moore, 2007). Many practitioners are now limiting what they prescribe and how much they prescribe despite the patients’ level of pain. This practice has affected the overall care that patients in pain now receive (Lin, Alfandre, & Moore, 2007).

Advanced practitioners are in a unique position to change clinical practice and the manner in which patients receive pain relief. They can coordinate pharmacologic and nonpharmacologic pain management strategies, assess barriers, develop new policies and procedures regarding pain management, and influence the way nurses practice and implement these methods They can also take a leading role in providing both formal and informal education to their nursing colleagues (Oncology Nursing Society, 2010).

## Limitations

The sample demonstrated a cross-sectional view of the knowledge of students who are currently pursuing a Bachelor of Science degree in nursing. Since the data were only collected from one geographic area and had limited representation from other ethnic groups, results gathered from this study may not be generalized to include the entire population of undergraduate nursing students in their state or the United States. As the sample mainly consisted of non-Hispanic white participants, the lack of representation from other ethnicities and cultures may have incurred a bias, with an unknown effect on data gathered; ethnicity and culture may influence knowledge and attitudes regarding pain management. Another limitation was the use of a convenience sample, which may have affected the data in some way.

## Conclusion

Although pain management has been an area of study for many decades, it is evident that a lack of student knowledge is a major hindrance in good pain management practices. Therefore, it can be surmised that better educating nurses and students is a step in the right direction toward optimal pain management practices. Hence, a change in the curricula for health care providers is required to improve current pain management practices. For this reason, topics addressing pharmacology and pain physiology in addition to better understanding of concepts such as tolerance, dependence, and addiction would be beneficial in improving the knowledge of students and creating better patient outcomes.

As leaders, APs can aid in this campaign by advocating for heightened pain management awareness in their roles as clinicians, educators, and researchers. Nursing education, which is mainly conducted by advanced-practice nurses who are on faculty, should focus on the development of specific strategies to effectively teach students about pain management as well as the integration of pain management content as a major component in the curriculum.

## Acknowledgments

The author would like to thank Cindy Tofthagen, PhD, ARNP, AOCNP®, for assisting with and sharing her invaluable insights during the preparation of this article.
